# Pancreatic cancer acquires resistance to MAPK pathway inhibition by clonal expansion and adaptive DNA hypermethylation

**DOI:** 10.1186/s13148-024-01623-z

**Published:** 2024-01-16

**Authors:** Laura K. Godfrey, Jan Forster, Sven-Thorsten Liffers, Christopher Schröder, Johannes Köster, Leonie Henschel, Kerstin U. Ludwig, David Lähnemann, Marija Trajkovic-Arsic, Diana Behrens, Aldo Scarpa, Rita T. Lawlor, Kathrin E. Witzke, Barbara Sitek, Steven A. Johnsen, Sven Rahmann, Bernhard Horsthemke, Michael Zeschnigk, Jens T. Siveke

**Affiliations:** 1 Bridge Institute of Experimental Tumor Therapy (BIT) and Division of Solid Tumor Translational Oncology (DKTK), West German Cancer Center, University Hospital Essen, University of Duisburg-Essen, Essen, Germany; 2 German Cancer Consortium (DKTK), partner site Essen, a partnership between German Cancer Research Center (DKFZ) and University Hospital Essen, Heidelberg, Germany; 3 Genome Informatics, Institute of Human Genetics, University Duisburg-Essen, Essen, Germany; 4 Bioinformatics and Computational Oncology, Institute for Artificial Intelligence in Medicine, University Hospital Essen, University Duisburg-Essen, Essen, Germany; 5 Institute of Human Genetics, School of Medicine & University Hospital Bonn, University of Bonn, Bonn, Germany; 6 EPO Experimental Pharmacology and Oncology GmbH, Berlin‐Buch, Germany; 7 Department of Diagnostics and Public Health, Pathological Anatomy Section, University and Hospital Trust of Verona, Verona, Italy; 8 ARC-Net Cancer Research Centre, University and Hospital Trust of Verona, Verona, Italy; 9 Medizinisches Proteom-Center/Zentrum Für Protein-Diagnostik, Ruhr-Universität Bochum, Bochum, Germany; 10 Department of General, Visceral and Pediatric Surgery, University Medical Center Göttingen, Göttingen, Germany; 11 Robert Bosch Center for Tumor Diseases, Stuttgart, Germany; 12 Algorithmic Bioinformatics, Center for Bioinformatics Saar and Saarland University, Saarland Informatics Campus, Saarbrücken, Germany; 13 Institute of Human Genetics, University Hospital Essen, University Duisburg-Essen, Essen, Germany; 14 National Center for Tumor Diseases (NCT) West, Campus Essen, Essen, Germany; 15 Klinik für Anästhesiologie, Intensivmedizin und Schmerztherapie, Universitätsklinikum Knappschaftskrankenhaus Bochum, Bochum, Germany

**Keywords:** Cancer, Therapy resistance, Epigenetic plasticity, DNA methylation, WGBS, Clonal expansion, PDAC

## Abstract

**Background:**

Pancreatic ductal adenocarcinoma (PDAC) is an aggressive cancer with poor prognosis. It is marked by extraordinary resistance to conventional therapies including chemotherapy and radiation, as well as to essentially all targeted therapies evaluated so far. More than 90% of PDAC cases harbor an activating *KRAS* mutation. As the most common *KRAS* variants in PDAC remain undruggable so far, it seemed promising to inhibit a downstream target in the MAPK pathway such as MEK1/2, but up to now preclinical and clinical evaluation of MEK inhibitors (MEK_i_) failed due to inherent and acquired resistance mechanisms. To gain insights into molecular changes during the formation of resistance to oncogenic MAPK pathway inhibition, we utilized short-term passaged primary tumor cells from ten PDACs of genetically engineered mice. We followed gain and loss of resistance upon MEK_i_ exposure and withdrawal by longitudinal integrative analysis of whole genome sequencing, whole genome bisulfite sequencing, RNA-sequencing and mass spectrometry data.

**Results:**

We found that resistant cell populations under increasing MEK_i_ treatment evolved by the expansion of a single clone but were not a direct consequence of known resistance-conferring mutations. Rather, resistant cells showed adaptive DNA hypermethylation of 209 and hypomethylation of 8 genomic sites, most of which overlap with regulatory elements known to be active in murine PDAC cells. Both DNA methylation changes and MEK_i_ resistance were transient and reversible upon drug withdrawal. Furthermore, MEK_i_ resistance could be reversed by DNA methyltransferase inhibition with remarkable sensitivity exclusively in the resistant cells.

**Conclusion:**

Overall, the concept of acquired therapy resistance as a result of the expansion of a single cell clone with epigenetic plasticity sheds light on genetic, epigenetic and phenotypic patterns during evolvement of treatment resistance in a tumor with high adaptive capabilities and provides potential for reversion through epigenetic targeting.

**Supplementary Information:**

The online version contains supplementary material available at 10.1186/s13148-024-01623-z.

## Background

Overcoming treatment resistance is a critical challenge for improving the prognosis for patients with pancreatic ductal adenocarcinoma (PDAC). Genetically, only four non-targetable genes (*KRAS*, *TP53*, *CDKN2A*, *SMAD4*) are known to be recurrently mutated in PDAC so far. Many low frequency alterations found in various genes reflect a remarkable inter- and intra-individual tumor heterogeneity [1–3]. Despite major advances in defining the genomic landscape of PDAC, the inherent and acquired resistance mechanisms during tumor evolution and upon therapeutic perturbations remain a considerable challenge.

Oncogenic *KRAS* mutations represent a therapeutic target in PDAC. Although inhibitors directed against specific oncogenic KRAS mutations such as KRAS G12C and G12D as well as newly developed pan-RAS inhibitors are recent exciting developments in the therapeutic armamentarium, their clinical benefit in PDAC remains to be proven. Potent and specific inhibitors for the downstream effector mitogen-activated protein kinase (MAPK) signaling pathway exist, including highly selective inhibitors against MEK, a component of the MAPK pathway [4]. However, despite promising results in preclinical model systems [5–7], MEK inhibitors (MEK_i_) have failed in clinical trials due to rapid induction of resistance [8–11]. Several cellular processes including mitochondrial function, nucleotide synthesis, protective autophagy or the deregulation of YAP, SHP or ERBB have been reported to be involved in MEK_i_ resistance [12–19]. However, their clinical relevance and the underlying regulatory circuits still remain to be identified.

Growing evidence supports a concept where tumor cells utilize epigenetic mechanisms to adapt to varying conditions, including MEK_i_ treatment of PDAC [20–22]. Mutations affecting epigenetic readers and writers such as enzymes controlling histone modifications and DNA methylation are frequently found in PDAC and other cancers [1, 23, 24].

In this study, we focus on longitudinal characterization of molecular alterations underlying MEK_i_ resistance. We combine multi-omics technologies on the genetic, epigenetic, transcriptomic and protein levels in primary genetically engineered mouse model (GEMM)-derived PDAC cells, thereby minimizing inter-individual genetic and epigenetic heterogeneity typically confounding patient-derived tumor analyses. We address adaptive epigenetic changes under therapeutic pressure and identify a vulnerability of MEK_i_-induced resistant PDAC cells to DNA methyltransferase inhibitors (DNMT_i_). We found adaptive DNA hypermethylation in cells that acquired MEK_i_ resistance and characterized its dynamics upon drug withdrawal.

## Results

### MEK_i_ resistance in PDAC is reversible upon drug withdrawal

To model MEK_i_ resistance in PDAC, we used primary low-passage cells derived from spontaneous PDAC of ten different *Ptf1a*^*wt/Cre*^*; Kras*^*wt/LSL−G12D*^*; Trp53*^*loxP/loxP*^ mice, which develop aggressive and therapy-resistant tumors resembling key aspects of human PDAC [25, 26].

**Fig. 1:**
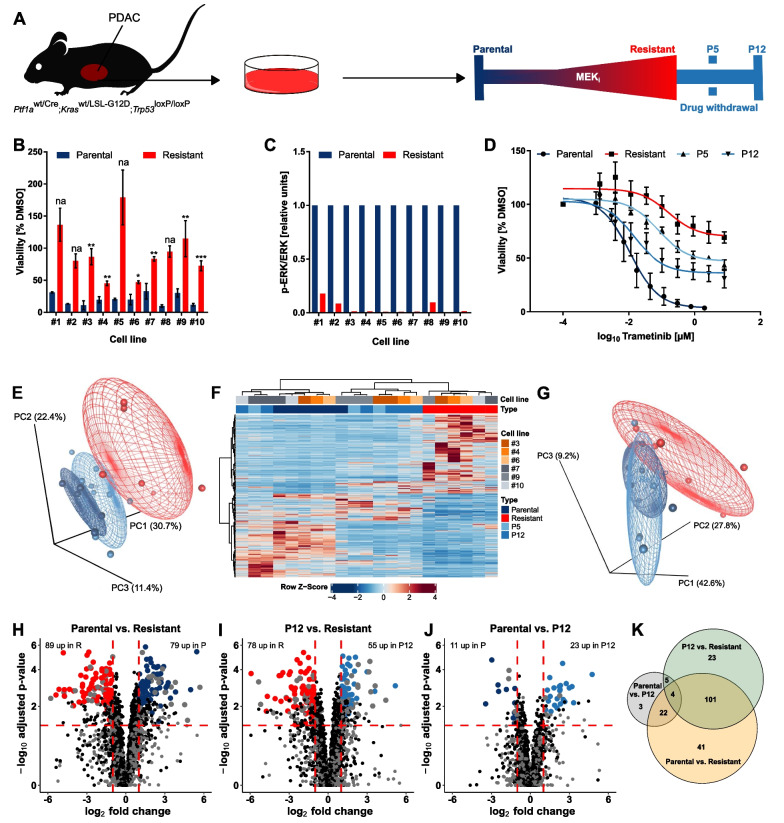
Acquired MEK_i_ resistance is reversible after drug withdrawal. **A** Timeline of resistance formation and drug withdrawal classified by different passages (P) without constant MEK_i_ treatment. **B** Bars represent the mean of two (na = two replicates in parental, three in resistant) or three independent cell viability measurements after 72 h in 300 nM MEK_i_ ± standard deviation (SD). DMSO controls were performed for both the parental and the resistant cells and used for normalization. Statistics was calculated by a two-tailed unpaired Student’s t test on the log_2_ transformed DMSO-normalized values. **C** ERK phosphorylation in parental compared to MEK_i_ resistant cell states assessed by Simple Western analysis (*p* < 0.0001, two-tailed paired Student’s t test on the log_2_ transformed ratios). The respective electropherograms and the derived recalculated images are shown in Additional file 2: Figs. S2 and S3. **D** Resistance reversibility upon drug withdrawal. The mean of three independent experiments after 72 h incubation ± SD is shown for cell line #3 as representative example. **E** Principal component analysis of RNA-seq data between parental (dark blue), resistant (red) and reverting (P5 and P12; light blue) cell states. Depictions in 2D are displayed in Additional file 2: Fig. S3. **F** Hierarchical clustering of significantly differentially expressed genes (adjusted *p* value < 0.01; log2 fold change > 1 or < − 1) between parental versus the union of resistant, P5, P12 and resistant versus the union of parental, P5, P12. **G** Principal component analysis of all abundances identified by LC-MS with more than one unique peptide. 2D presentations are shown in Additional file 2: Fig. S3. **H**–**J** Differentially expressed proteins determined by LC-MS between resistant and parental (**H**) or P12 (**I**) as well as between parental and P12 cells (**J**). Larger dots (blue and red) indicate significance (FDR < 0.05, log2 fold change < -1 or > 1) in ANOVA and post hoc test between the indicated groups and at least 2 unique peptides. Gray dots represent abundances only identified by 1 unique peptide. **K** Euler diagram summarizing the mass spectrometry results. Numbers indicate significantly different abundances of proteins per comparison identified with more than 1 unique peptide

All primary cells lines (n = 10) were sensitive to MEK_i_ with an IC50 in the low nanomolar range (5.44 nM to 41.91 nM, median 12.70 nM) (Additional file 3: Table S2). To induce MEK_i_ resistance, cells were treated with increasing trametinib doses over 3 to 4 months until they proliferated at 100-fold of the original IC50 dose (Fig. 1A). Resistance induction was successful in all 10 cell lines (Fig. 1B and Additional file 2: Fig. S1) and was accompanied by a strong block of ERK phosphorylation, underpinning the specificity of trametinib and suggesting a drug efflux independent resistance mechanism (Fig. 1C and Additional file 2: Figs. S2 and S3). To study the effect of drug withdrawal, one batch of resistant cells from each line was cultivated without MEK_i_ and samples were collected after 5 (P5) and 12 passages (P12) of drug withdrawal (Fig. 1A). Thereby a reversibility of the resistant phenotype was observed correlating with the duration of drug-free time (Fig. 1D).

We compared the expression profiles associated with acquired MEK_i_ resistance by performing RNA-sequencing (RNA-seq) of six matched parental, resistant and reverting cell states. Results of principal component analysis (PCA) over all expressed genes showed that the reverting states (P5 and P12) had a transcriptional profile more similar to the parental cells (Fig. 1E and Additional file 2: Fig. S4A,B). The inter-individual variability between cell lines explained a considerably smaller proportion of the transcriptional variation than treatment. We then performed supervised hierarchical clustering based on genes differentially expressed in parental or resistant cells (Fig. 1F). The resulting dendrogram showed that some reverting cells clearly grouped with the parental cells while the expression signature of other reverting cells was more similar to the resistant cells. A similar grouping could be observed on the protein level measured by mass spectrometry (Fig. 1G and Additional file 2: Fig. S4C,D and Additional file 1: Table S3). Concordantly, 168 proteins were significantly differentially expressed between parental and resistant cells, while only 34 proteins differed between parental and P12 cells (Fig. 1H,I). Furthermore, 105 of the 168 differently expressed proteins between parental and resistant cells also varied between P12 and resistant cells, thus supporting the reversibility of the MEK_i_ resistant phenotype not only on the mRNA but also on the protein level (Fig. 1J,K).

### MEK_i_ resistance is characterized by EMT and a PDAC subtype switch

**Fig. 2:**
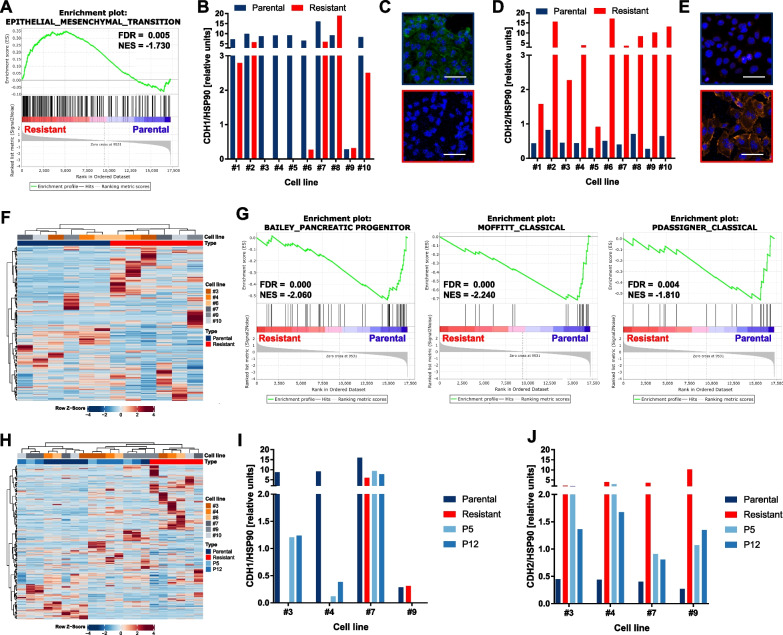
MEK_i_ resistance is accompanied by a phenotypic switch. **A** GSEA of RNA-seq from parental compared to resistant cells revealed enriched hallmarks of EMT. **B**–**E** Significantly decreased CDH1 (**B**) and increased CDH2 (**D**) protein expression assessed by Simple Western (*p* < 0.05, two-tailed paired Student’s t test on the log2 transformed ratios) and corresponding immunofluorescence of one representative example (cell line #3) (**C, E**). Either CDH1 (green) or CDH2 (orange) were stained together with DAPI (blue) nuclear staining (scale bars: 50 μm). **F** Hierarchical clustering of parental and resistant cells based on the metagenes for the pancreatic progenitor and the squamous subtype as defined by Bailey et al. [27]. **G** GSEA of metagenes defining the pancreatic progenitor or the classical subtype as defined by Bailey et al. [27] (left), Moffitt et al. [29] (middle) or Collisson et al. [28] (right). **H** Hierarchical clustering like in (F) including P5 and P12 samples. **I, J** Normalized CDH1 (**I**) or CDH2 protein expression (**J**) in four matched parental, resistant, P5 and P12 samples measured by Simple Western, respectively

Global gene expression profiling revealed that resistant cells were significantly enriched (false discovery rate (FDR) = 0.005, normalized enrichment score (NES) = 1.73) for transcripts involved in epithelial-mesenchymal transition (EMT) indicating a phenotypic switch from an epithelial to a more mesenchymal cell state (Fig. 2A). On the protein level, we found a decreased expression of the epithelial marker CDH1 (Fig. 2B,C and Additional file 2: Fig. S5) and a strong increase of the mesenchymal marker CDH2 (Fig. 2D,E and Additional file 2: Fig. S6) confirming EMT specific features of the resistant cells. We performed hierarchical clustering based on the expression of known PDAC subtype-specific genes [27–29]. Using the gene set defined by Bailey et al. [27], the parental cells were clearly separated from the resistant cells (Fig. 2F). Using gene sets defined by Collisson et al. [28] and Moffitt et al. [29], parental cells of line #9 grouped together with the resistant cells (Additional file 2: Fig. S7A,B). Concordantly, cell line #9 showed the highest IC50 and was, based on its lack of CDH1 already in the parental state, the most mesenchymal line in our cohort, which highlights the association of MEK_i_ resistance with a mesenchymal phenotype.

We defined gene sets based on the mentioned subtype-specific genes and performed Gene Set Enrichment Analysis. All three classical/pancreatic progenitor subtype-defining gene sets were significantly (FDR < 0.001, NES < − 1.8) enriched in the parental cells (Fig. 2G).

Including the reverting cell states P5 and P12 in the cluster analysis based on the Bailey et al. [27] metagenes, the clear separation of parental and resistant cells (except for line #9 as mentioned) remained unchanged (Fig. 2H). In the cases where RNA of P5 and P12 derived from the same cell line was sequenced, the two samples clustered together. The reverting samples either built a separate cluster adjacent to the parental or the resistant samples indicating transcriptional changes in the subtype-defining genes after drug withdrawal. Similar results were obtained by cluster analyses using the Moffitt et al. [29] or Collison et al. [28] data sets (Additional file 2: Fig. S7C,D). Furthermore, the protein expression of CDH1 recovered in P5 and P12 apart from cell line #9 which had a poor CDH1 expression even in the parental cells (Fig. 2I). In contrast, the expression level of the mesenchymal-associated protein CDH2 decreased in the absence of MEK_i_ in the culture medium (Fig. 2J).

Taken together, our data support a phenotypic switch correlating with MEK_i_ susceptibility and the presence or absence of the drug in the culture medium. Resistant cells in the presence of MEK_i_ seem to be most mesenchymal.

### Whole genome sequencing-based mutation analysis of MEK_i_ resistant cells

**Fig. 3:**
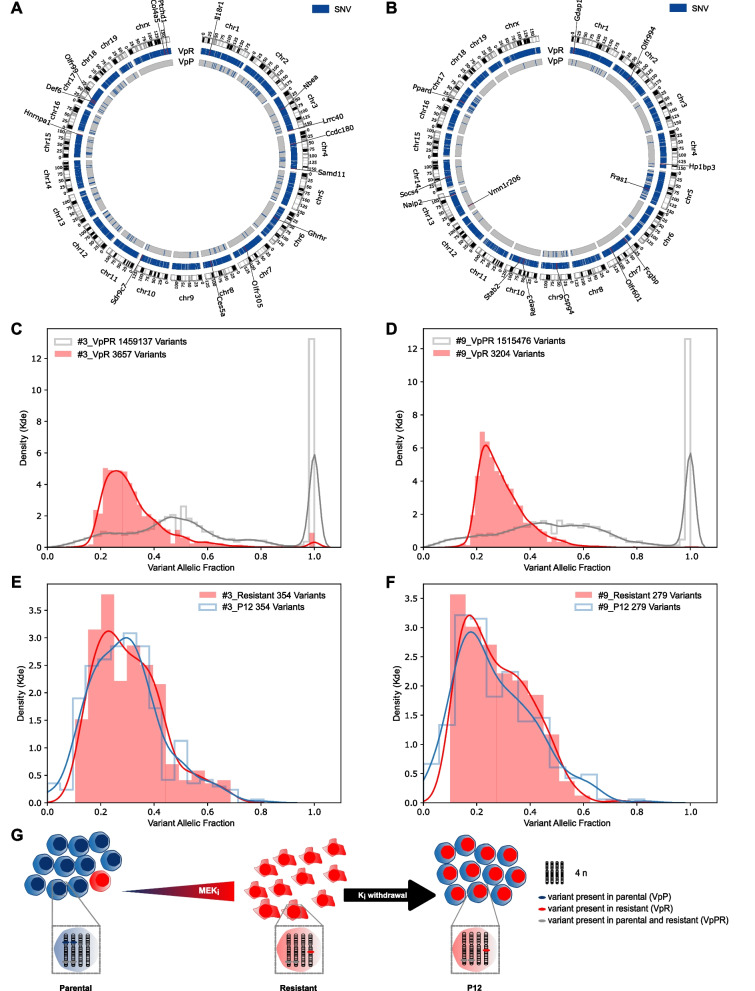
MEK_i_ resistance is based on clonal expansion, while reversion upon drug withdrawal is independent of a parental outgrowth. **A, B** Circos plots displaying VpRs or VpPs in cell lines #3 (**A**) and #9 (**B**). Genes predicted as deleterious by SIFT are named. **C, D** Kernel density estimation (kde) for the VAF of VpRs in comparison with variants present in parental and resistant (VpPRs) for cell line #3 (**C**) or #9 (**D**). **E, F** Density plot for the VAF of VpRs in resistant compared to P12 in cell line #3 (**E**) or #9 (**F**). Only A > T and T > A variants called by WGS and validated by WGBS are shown. **G** Model of cell population dynamics during gain and loss of MEK_i_ resistance in PDAC

Genetic and non-genetic alterations may contribute to the development of a resistant phenotype [30]. To determine whether genetic alterations were associated with MEK_i_ resistance, we performed whole genome sequencing (WGS) of two matched pairs of parental and resistant cell states with a median coverage of 40x. Compared to their treatment-naïve counterparts, resistant cells of lines #3 and #9 harbored 3657 and 3204 unique single nucleotide variants (SNVs), respectively. These variants are referred to as ‘variant present in resistant’ (VpR) (Fig. 3A,B; Additional file 1: Tables S4 and S5). A smaller number of variants was present in the parental cells, but could not be detected in the resistant cells (‘variant present in parental’, VpP) (131 in cell line #3 and 837 in cell line #9). Less than 1% of the VpRs were located in the coding regions (CDS) (36 in #3 and 23 in #9) (Additional file 1: Tables S4 and S5). Of these, 25 (cell line #3) and 19 (cell line #9) were nonsense or missense mutations, respectively (Additional file 1: Tables S4 and S5).

SIFT prediction to assess the impact of these VpRs revealed 12 and 9 possibly deleterious variants for cell lines #3 and #9, respectively (Fig. 3A,B, Additional file 1: Tables S4 and S5). The putative variant effect supposed by Jannovar [31] was moderate for those classified as deleterious by SIFT. None of them were found in both cell lines nor affected the same genes. None of the genes affected by the VpRs are known to be involved in the RAS-dependent MEK_i_ targeted MAPK or in the phosphoinositide 3-kinase (PI3K) pathway. In accordance, none of the mutant genes was listed in the COSMIC database to confer therapy resistance to human cancer cells. In addition to SNVs, InDels were called. However, we found none of the genes affected to be involved in re-activation or bypassing of the MEK pathway (Additional file 1: Tables S4 and S5). Overall, we did not identify any genetic variant that might explain the resistant phenotype.

### Whole genome sequencing reveals clonal expansion during acquired MEK_i_ resistance

We next addressed whether MEK_i_ resistance evolves by expansion of a subclone from the parental cells. Therefore, the distribution of the allele fraction of all variants found in resistant cells only (VpRs) was analyzed revealing a variant allele fraction (VAF) peak at around 0.25 in both cell lines (Fig. 3C,D). A potential explanation is the presence of this SNVs on one allele on a genetic background of four alleles, e.g., in cells with tetraploidy as consequence of genome duplication. Polyploidization can be found in approximately 50% of human PDAC [32]. By performing a cytogenetic analysis of metaphase chromosomes, we found in median 77 and 70 chromosomes per cell in parental cells of lines #3 and #9, respectively, which is in concordance with a near tetraploid karyotype (Additional file 3: Table S6). Evaluation of the VAF per chromosome revealed a lower chromosome count for chromosomes 9, 12 or 13 of cell line #3 or chromosomes 5 or 19 of #9 with VAF peak > 0.25, (Additional file 2: Fig. S8A,B). Taken together, the observed VAF distribution peak at 0.25 in these nearly tetraploid cells suggests that the cells with acquired MEK_i_ resistance are the result of clonal expansion of a single cell clone. Furthermore, most VpRs must have occurred after the incomplete genome duplications.

We next investigated whether a small proportion of parental cells that do not carry the VpRs might have survived the MEK_i_ treatment and then overgrown the VpR-containing resistant cells upon drug withdrawal. Therefore, we evaluated the VpR and VpR allele fractions in whole genome bisulfite sequencing (WGBS) data available for parental, resistant and reverting (P12) cells. Due to cytosine conversion by bisulfite modification, only a subset of 354 or 279 VpRs and 10 or 36 VpPs were available for further evaluation in cell lines #3 and #9, respectively. Nearly all VpRs were present in P12 cells with a VAF distribution similar to the resistant cells (Fig. 3E–G and Additional file 2: Fig. S8C,E). Furthermore, all but 2 VpPs were absent in P12 cells (Additional file 2: Fig. S8D,F). Thus, despite rebounding MEK_i_ susceptibility, the resistant genotype persists in P12 cells.

Overall, MEK_i_ resistance is based on clonal expansion of a single cell clone, without evidence for mutations or structural variation in genes that could mediate MEK_i_-induced resistance. Loss of resistance upon drug withdrawal does not occur by outgrowth of parental cells.

### MEK_i_ resistance is associated with DNA methyltransferase inhibitor sensitivity

**Fig. 4:**
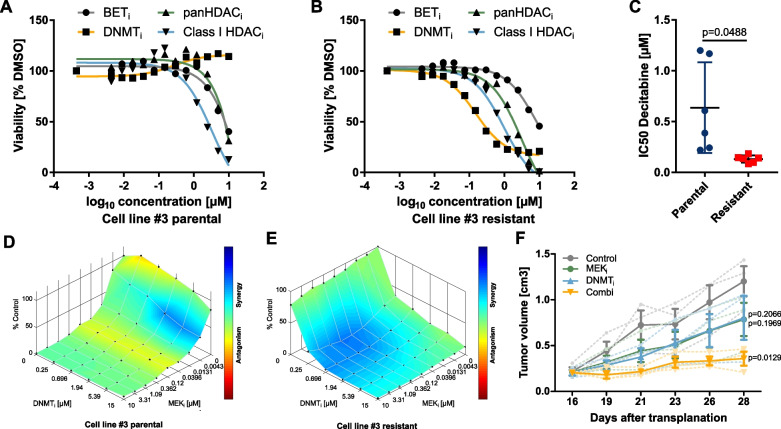
DNA methyltransferase inhibition effectively targeted MEK_i_ resistant cells only. **A, B** Cell viability screen using inhibitors that target different epigenetic mechanisms in parental (**A**) or resistant cells (**B**). Dose response curves for cell line #3 determined after 72 h using the CellTiter-Glo® cell viability assay are shown. **C** The IC50 of DNMT_i_ was significantly lower in six matched pairs of parental versus resistant cells (two-tailed paired Student’s t test). **D, E** Synergy analysis of MEK_i_ plus DNMT_i_ using the Loewe method of the Combenefit software shown for cell line #3 parental (**D**) and resistant (**E**). **F** Growth curves of PDX treated with MEK_i_, DNMT_i_ or the combination. Solid lines represent the mean tumor volume of three mice per treatment group ± standard error of the mean (SEM). Statistical significance versus control was determined using a two-tailed unpaired Student’s t test

To address the potential involvement of epigenetic mechanisms in transcriptional variability, we applied a targeted drug screening approach addressing key epigenetic regulators including chromatin readers, histone modifiers and DNA methyltransferases in MEK_i_-resistant cells. We found a strong effect of the DNMT_i_ decitabine on the viability of resistant but not parental cells (Fig. 4A–C). The difference was by far more pronounced compared to inhibitors of other epigenetic regulators such as bromodomain and extracellular terminal (BET) proteins, class I-specific or pan-histone deacetylases (HDAC), suggesting that MEK_i_-induced formation of resistance involves critical changes in DNA methylation (Fig. 4A,B). In resistant cells, a synergistic effect of MEK_i_ and DNMT_i_ was observed even at low doses of DNMT_i_. Synergism in the parental cells was only observed at high DNMT_i_ concentrations (Fig. 4D,E and Additional file 2: Fig. S9A−F).

In addition, we investigated the effect of MEK_i_ and DNMT_i_ inhibition using a human in vivo setting. We treated three different PDX models with either MEK_i_, DNMT_i_ or a combination of both and observed a synergistic effect in all three models (Fig. 4F and Additional file 2: Fig. S9G,H).

### MEK_i_ resistance is associated with DNA hypermethylation

**Fig. 5:**
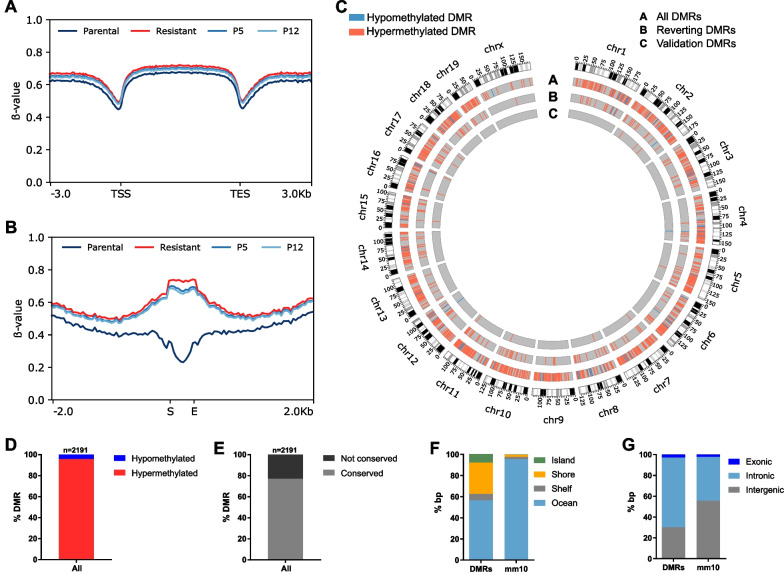
Whole genome bisulfite sequencing revealed DMRs mainly hypermethylated in MEK_i_ resistant cells. **A** Mean gene body methylation levels in parental, resistant, P5 and P12 cell states of cell lines #3 and #9. TSS indicates the transcription start site and TES the transcription end site. **B** Mean methylation levels of all DMRs and their flanking regions (± 2 kb) in the four indicated cell states of two different cell lines (#3, #9). S indicates the DMR start and E its end. **C** Circos plot indicates the chromosomal location of 2191 DMRs between two parental and resistant cell lines (circle A). A scoring function was developed to define 217 reverting DMRs whose methylation pattern in P12 resembles that of parental cells (circle B). Circle C displays 15 DMRs that were validated by targeted deep bisulfite sequencing. **D** Proportion of DMRs hypo- or hypermethylated in resistant cells. **E** Degree of mouse-human DMR-sequence conservation according to the UCSC liftover tool. **F, G** Relative location of DMRs on bp level in relation to CpG islands (**F**) or genes (**G**) both compared to the mm10 reference genome (*p* < 0.001; chi-square test)

To assess MEK_i_ treatment-induced alterations in genome-wide DNA methylation, we performed whole genome bisulfite sequencing of two cell lines, each at four different states: parental, resistant and reverting cells at P5 and P12. Overall gene body and promotor methylation levels between all four cell states remained unchanged (Fig. 5A). Here, we defined regions as differentially methylated (DMRs) if at least four consecutive cytosine-guanine dinucleotides (CpGs) showed a minimal difference in methylation level of 0.4 in both cell lines. Thereby, 2191 DMRs relatively equally distributed over all chromosomes were found when comparing parental and resistant cells (Fig. 5B,C and Additional file 1: Table S7). These DMRs covered a total of 38,031 CpGs with a mean CpG content per DMR of 17 (min = 4, max = 178) and an average length of 794 bp (min = 12 bp, max = 4157 bp) (Additional file 1: Table S7). Remarkably, more than 96% of these DMRs were hypermethylated in the resistant cells (Fig. 5D). The nucleotide sequence of the majority of 1756 DMRs was conserved in humans based on the UCSC liftover tool corresponding to a degree of conservation > 76% (Fig. 5E and Additional file 1: Table S7). 43.37% were located in ocean areas of the genome, while 39.25% and 6.12% were present in the CpG island flanking shores and shelves, respectively. Only 8.26% of all DMRs overlapped with a CpG island (Fig. 5F). However, the association of CpG island-related regions was significantly higher in the DMRs compared to the mm10 reference genome (*p* < 0.001; chi-square test). Approximately one third (29.53%) of the DMRs was located in intergenic regions, while the others were present in intragenic, predominantly intronic regions (65.95%) (Fig. 5G). The intragenic fraction in the DMRs was significantly higher than in the whole mm10 reference genome (*p* < 0.001; chi-square test).

In summary, we identified extensive hypermethylation following acquired MEK_i_ resistance, supporting epigenetic plasticity in our model system.

### A DMR subset reverts upon MEK_i_ withdrawal

**Fig. 6:**
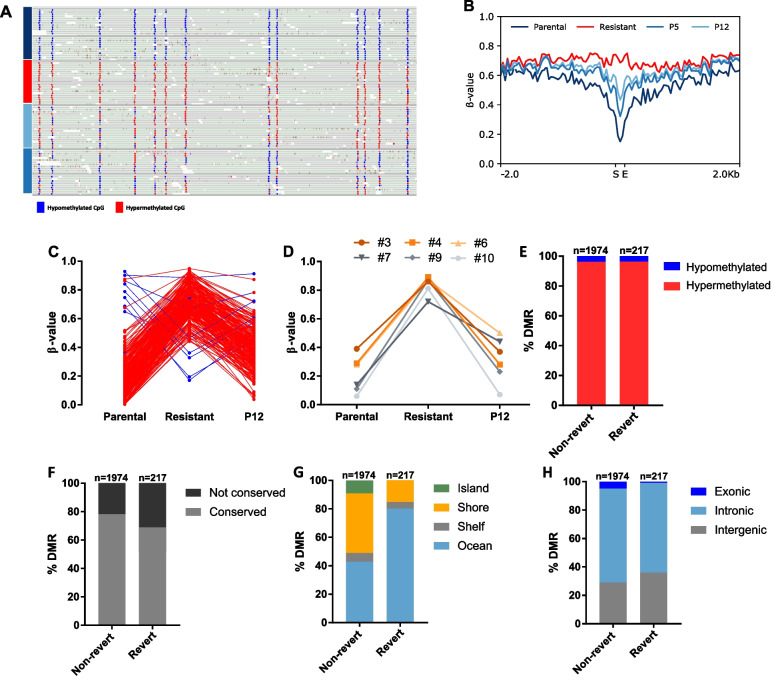
Distinct DMRs revert after MEK_i_ withdrawal **A** Representative Integrative Genomics Viewer snapshot of reverting DMR_1716 in the parental (dark blue), resistant (red), P5 (light blue) and P12 (blue) cell states of cell lines #3 and #9 (upper row and lower row, respectively). 13 of 16 CpGs are shown. **B** Mean methylation levels of the reverting DMRs and their 2 kb up- and downstream regions of cell lines #3 and #9 in the parental, resistant, P5 and P12 cells (S: Start DMR; E: End DMR). **C** Methylation pattern of 217 reverting DMRs divided into hypo- (blue) and hypermethylated (red) regions. **D** Methylation pattern of DMR_929 in four independent cell lines compared to the WGBS samples based on the average methylation b-value in the region. **E**–**H** Comparison of reverting and non-reverting DMRs based on methylation change in resistant cells (**E**), sequence conservation in human (**F**) and their relative location to CpG islands (**G**) or genes (**H**)

We next addressed the question if DMRs involved in MEK_i_ resistance may revert upon drug withdrawal (Fig. 6A). We identified 217 DMRs, from here on referred to as reverting DMRs, whose methylation status correlated with MEK_i_ sensitivity at all stages analyzed (Fig. 6B,C and Additional file 1: Table S7). This correlation was further supported by the observation that the degree of methylation in P5 cells with intermediate resistance phenotype was always between the value of the resistant cells and the P12 cells (Additional file 2: Fig. S10A,B).

We validated a set of 15 selected DMRs in four additional cell lines in parental, resistant and P12 states using targeted deep amplicon bisulfite sequencing [33]. Ten out of 15 DMRs were differentially methylated between parental and resistant cells in at least two of four cell lines used for validation. In addition, eight of these 10 DMRs showed a reverting DNA methylation in the P12 state (Fig. 6D and Additional file 1: Fig. S11). In particular, the DNA methylation levels of reverting DMRs in cell lines #3 and #9 remained at P12 levels or below even after 40 passages under MEK_i_ withdrawal (Fig. 6E and Additional file 2: Fig. S11).

Comparing various features of reverting and non-reverting DMRs, we found the proportion of hypomethylated DMRs to be the same in both groups (Fig. 6F). The number of human-mouse conserved DMRs was slightly lower in the reverting DMRs (Fig. 6G). Reverting DMRs were more frequently located in the ocean than non-reverting DMRs (80.18% vs. 42.65% in non-reverting DMRs), while they were underrepresented in shores (Fig. 6H). Notably, none of the reverting DMRs overlapped with a CpG island. In exonic regions, reverting DMRs were less frequent (0.92%) compared to non-reverting DMRs (4.91%) (Fig. 6I).

### In silico evaluation of regulatory relevance of reverting DMRs

**Fig. 7:**
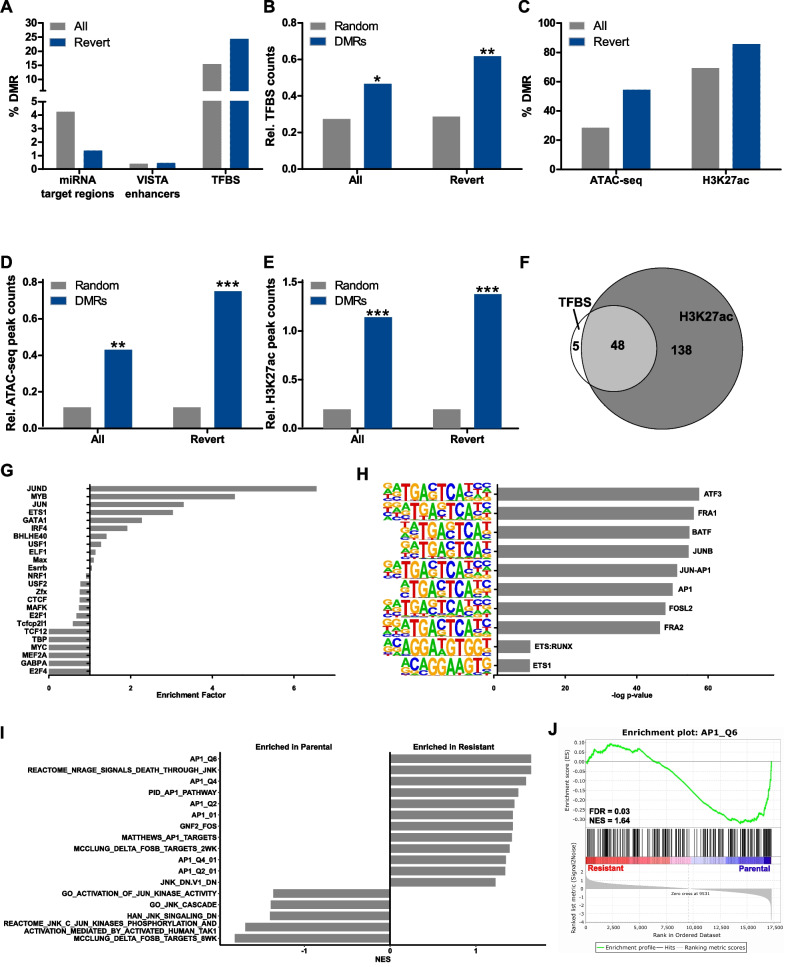
Functional relevance of DMRs and DNA methylation in MEK_i_ resistance. **A** Percent of DMRs overlapping with the indicated feature. **B** TFBS were significantly enriched in all as well as in reverting DMRs compared to 10^6^ random regions of similar length and CpG count (** significance level < 0.01; * significance level < 0.05). **C** Percent of DMRs that overlap with an ATAC-seq or ChIP-seq H3K27ac peak found in at least 2 organoids by Roe and co-workers [34]. **D, E** Enrichment for ATAC-seq (**D**) or H3K27ac (**E**) peaks relative to 10^6^ random regions of similar length and CpG count (*** significance level < 0.001; ** significance level < 0.01). **F** Venn diagram of reverting DMRs’ overlap with TFBS and/or H3K27ac peaks. **G** Enrichment of TFBS for the indicated transcription factors in the 217 reverting DMRs compared to 1974 non-reverting DMRs. **H** Enrichment of TFBS motifs from the homer database between the reverting DMRs and random background sequences. **I** NES of AP1 related gene expression signatures that are significantly (FDR < 0.25) different between parental and resistant cells based on GSEA of RNA-seq data. **J** Enrichment plot of the AP1_Q6 gene expression signature

To investigate the potential relevance of the reverting DMRs, we analyzed their co-localization with microRNA (miRNA) target regions, VISTA enhancers and transcription factor binding sites (TFBS) as annotated by Ensembl. (Fig. 7A and Additional file 1: Table S7). Compared to randomly selected regions with similar length and CpG content, only TFBS were significantly enriched in all 2191 DMRs and even more pronounced in the 217 reverting DMRs (Fig. 7B).

The activity of regulatory elements is highly tissue- as well as context-specific and their deregulation is often observed in cancer. Therefore, we aligned the identified DMRs with sequences reported to represent open chromatin (assay for transposase-accessible chromatin and sequencing; ATAC-seq) or potential active enhancer sites (chromatin immunoprecipitation and sequencing (ChIP-seq) for H3K27ac) in murine pancreas cells [34]. These data were obtained from murine organoids, similar to our model, derived from PDAC of KPC mice (*Kras*^*wt/LSL−G12D*^*; Trp53*^*wt/LSL−R172H*^*; Pdx1-Cre*). We found that 28.5% of all 2191 DMRs overlapped with ATAC-seq peaks, which comprise only about 0.8% of the genome, while 3% of the genome, but 69.3% of all DMRs overlapped with H3K27ac ChIP-seq peaks (Fig. 7C and Additional file 1: Table S7). Thus, DMRs were significantly enriched for open and/or active (H3K27ac histone occupied) chromatin regions compared to random regions of similar length and CpG content (Fig. 7D,E). In both cases, this enrichment was even more pronounced for reverting DMRs. More than 90% of TFBS-containing reverting DMRs overlapped with a H3K27ac-marked region, which underlines their potential regulatory relevance in MEK_i_-resistant PDAC cells (Fig. 7F).

Using two independent enrichment analysis tools, we found that binding motifs for proteins belonging to the activator protein 1 (AP1) family were among the top enriched TFBS (Fig. 7G,H). It is well documented that AP1 binding to its respective motif is strongly dependent on the methylation status of its recognition site and proximity [35, 36]. AP1 is a protein dimer formed by members of the JUN, FOS and ATF families. Notably, JUN was among the proteins regulated in MEK_i_-treated PDAC cells. Resistant cells displayed both an increased total protein expression and enhanced JUN phosphorylation at Ser73 (Additional file 2: Figs. S12 and S13). Furthermore, different AP1/JUN expression signatures were enriched in the parental cell lines (Fig. 7I,J).

In conclusion, MEK_i_ resistance is associated with DNA hypermethylation at regulatory elements including TFBS and active enhancer sites located in open chromatin. We identified the AP1 transcription factor complex as a potentially crucial factor in mediating MEK_i_-induced resistance. Overall, our data show a key relevance of DNA methylation for maintaining MEK_i_ resistance in PDAC, which results in a remarkable DNMT_i_ vulnerability.

### Discussion

PDACs are characterized by a remarkable resistance to virtually all therapeutic strategies. Several aspects challenge the unraveling of underlying mechanisms [37]. A key limitation of studies in advanced PDAC is the lack of longitudinal sample acquisition in individual treated patients due to anatomical, ethical, and logistical reasons among others.

Here, we utilized a cell culture model based on low-passage primary cells derived from tumors of a genetically engineered PDAC mouse model [25] to analyze molecular changes that arise under MEK inhibition. The use of tumor cell populations with the same genetic background minimizes the influence of confounding factors such as inter- individual heterogeneity or contaminating non-tumor cells, the latter being a major concern when using primary PDAC tumor tissue. Consequently, our model cannot account for non-tumor cell intrinsic mechanisms such as involvement of the tumor microenvironment [38]. However, the observation that we were able to induce MEK_i_ resistance in all ten cell lines suggests that, at least in our model system, resistance formation is mediated by tumor cell intrinsic mechanisms.

Using WGS, we clearly demonstrate that the resistant cell clones evolve from a single precursor cell in the parental cell population. It is not possible to distinguish whether the genetic variants observed in the resistant cells were already present in the parental cells or arose during MEK_i_ treatment or a combination of both. The observed clonality strongly indicates that the parental cell population is composed of cells with different abilities to adapt to MEK_i_ treatment with single cells having the potential to develop a resistance phenotype.

In a human PC-9 lung cancer model, Hata et al. [39] compared gefitinib resistant cells harboring a resistance-mediating *EGFR* mutation with resistant cells that expanded under treatment without such mutations. Gefitinib-resistant *EGFR* mutant PC-9 cells had a similar transcriptional profile to their parental gefitinib-naive cell pool [39]. In contrast, transcriptomic differences found between our mouse parental and MEK_i_- resistant cells were also described for PC-9 cells without *EGFR* resistance mutation [39]. Furthermore, it took about four months from the onset of drug exposure to a fully MEK_i_-resistant cell population [39]. This is a similar time frame to that observed for the PC-9 lung cells without a known resistance-mediating *EGRF* mutation [39]. Whereas PC-9 cell pools harboring an *EGFR* resistance-mediating mutation, developed resistance within 6 weeks [39]. Given the similar proliferation rates of cells in our PDAC mouse model and the PC-9 cells, this argues against a mutation in a classical resistance gene as underlying cause of MEK_i_ resistance. Consistently, using WGS we did not detect mutations in genes involved in re-activation or bypassing of the targeted MAPK pathway in the resistant cells. Although this approach cannot definitively rule out the presence of such mutations, it provides clues for the presence of alternative mechanisms that confer MEK_i_ resistance to cells. This is further supported by the observation that cells lose their resistance phenotype during drug withdrawal without re-gaining the parental genotype. As it is unlikely that the formation of resistance is a direct consequence of mutations in genes involved in drug resistance, we hypothesize that the predisposition of the originating cell could be due to an epigenetic plasticity that enables it to adapt better to the environmental conditions than other cells, e.g., by DNA methylation changes. Whether this plasticity is the consequence of sequence alterations already present in the parental cell or due to stochastic epigenetic variation remains to be determined. An example for an epigenetic factor contributing to phenotypic adaptation upon drug treatment is described in a study by Wang et al. [21] showing that modulation of histone methylation is involved in MEK_i_ resistance.

Our hypothesis of a cell clone capable of epigenetically adapting to MEK_i_ treatment is further supported by the association of transcriptional plasticity with MEK_i_ resistance. The resulting EMT and the switch to a more mesenchymal phenotype reflect the quasi-mesenchymal/basal-like subtype of PDAC [27–29]. EMT is known to be linked to stemness that enables tumor cells to develop drug resistance [39, 40]. Cells with mesenchymal properties have been reported to be less sensitive to MEK_i_ treatment than epithelial ones43. It is tempting to speculate that, these properties allow cells a comprehensive modulation of their methylome, which normally only occurs during development or cell differentiation.

As DNA methylation is an important epigenetic mediator already known to be involved in the progression of PDAC or other tumors, as well as the formation of distant metastases, we determined DNA methylation changes in our PDAC model [41]. To our knowledge, we provide the first WGBS dataset of therapy-resistant PDAC, albeit in murine cells, which provides an unbiased and comprehensive view at the dynamic changes of the methylome in cells in response to drug exposure and subsequent drug withdrawal. The differentially methylated regions showed a high degree of conservation between the mouse and human genome and overlapped with known regulatory elements like TFBS and potential enhancer sites, which supports their functional relevance in gene regulation. We could also identify a subset of reverting DMRs whose gain and loss of methylation reflected gain and loss of the resistant phenotype. Among these reverting DMRs, binding sites for the dimeric transcription factor complex AP1 were significantly enriched. Its DNA hypermethylation, as present in the resistant cell states, is known to prevent AP1 binding [35, 36].

It is a matter of debate if DNA methylation changes are causally involved in the regulation of gene expression or if they develop downstream of transcription factor- mediated gene regulation [42]. Interestingly, the DNA methylation changes that occur during drug exposure and resistance formation are almost exclusively DNA hypermethylation events. Such de novo methylation of previously unmethylated CpGs depend on the activity of methyltransferases DNMT3A und DNMT3B. Therefore, blocking de novo methylation could prevent or impede the formation of MEK_i_ resistance in PDAC cells or primary tumors. Indeed, the synergistic effect of combined MEK_i_ and DNMT_i_ treatment observed in resistant PDAC cells strongly suggest that MEK_i_ resistance is attenuated by decitabine treatment. Decitabine is an inhibitor that blocks the activity of both de novo methyltransferases and of the maintenance methyltransferase DNMT1. It will be interesting to evaluate if drug resistance in general is associated with DNA hypermethylation or if this observation is restricted to MEK inhibitors in PDAC cells.

#### Conclusions

**Fig. 8:**
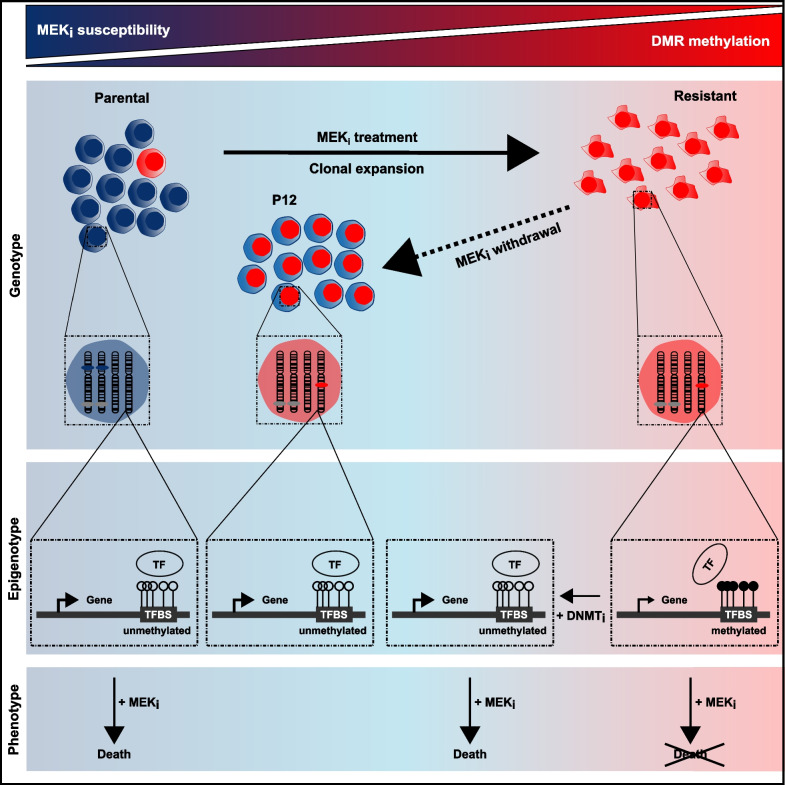
Model of cell population dynamics and associated cellular alterations in MEK_i_ resistance of PDAC. (TF: transcription factor, TFBS: transcription factor binding site)

Overall, our results of a MEK_i_ adaptive DNA hypermethylation landscape of regulatory regions in a single cell clone support epigenetic plasticity of tumor cells as a driver in PDAC therapy resistance (Fig. 8). The remarkable DNMT_i_ sensitivity of the MEK_i_ resistant cells might inspire new combinatory therapeutic approaches to overcome therapy resistance in PDAC.

## Methods

### Generation of primary murine PDAC cell lines

Tumor pieces derived from *Ptf1a*^*wt/Cre*^*; Kras*^*wt/LSL−G12D*^*; Trp53*^*loxP/loxP*^ mice were incubated at 37°C and 5% CO_2_ in high-glucose Dulbecco's Modified Eagle's Medium (DMEM) (Thermo Fisher Scientific) containing 10% fetal bovine serum (Thermo Fisher Scientific), 1% penicillin/streptomycin (Thermo Fisher Scientific), and 1% non-essential amino acids (Sigma-Aldrich) until tumor cells emigrated. PCR-based mycoplasma testing was performed on a regular basis. Cell lines #1, #3, #4, #5 and #8 were derived from male mice and cell lines #2, #6, #7, #9 and #10 from female mice. All cell lines used are available from the corresponding author upon reasonable request.

### MEK_i_ resistance induction in primary murine PDAC cell line

MEK_i_ resistance was induced in ten different low-passage cell lines (< 4–12 passages). Therefore, cells were treated with increasing doses of trametinib (LKT) until they grew in 100 × of their IC50 (800 nM to 4200 nM trametinib). One batch of each cell line was cultivated with 100 × IC50 of trametinib in the culture medium (termed resistant hereafter) with medium exchange every 2–3 days on a regular basis. Another batch was kept under drug withdrawal and samples were named according to their passage number in drug-free medium (Px).

### Cell viability assays

Cell viability assays were performed with four to six different cell lines. Cell numbers were optimized for 80% confluency in 96- or 384-well plates, respectively. Drugs targeting different epigenetic modifiers (trametinib (LKT), decitabine (Sigma-Aldrich), JQ1 (Cayman), suberoylanilide hydroxamic acid (SAHA) (Selleckchem), mocetinostat (Selleckchem) dissolved in dimethyl sulfoxide (DMSO) (Sigma-Aldrich) were printed in the indicated logarithmic concentration ranges using the D300e Digital Dispenser (Tecan). The DMSO concentration in each well was adjusted to the highest value on the plate which was set to < 0.1% of the assay volume. Sealed plates were frozen at − 80 °C until use.

Cells were detached by 0.05% trypsin—ethylenediamine tetraacetic acid (EDTA) (1x) (Thermo Fisher Scientific) and recovered by centrifugation. Optimized cell numbers for 80% confluency at the end of experiment were seeded with the Multidrop Combi Dispenser (Thermo Fisher Scientific) onto the pre-printed plates and incubated at 37°C and 5% CO_2_.

Cell viability was determined using the CellTiter-Glo® Luminescent Cell Viability Assay (Promega) according to manufacturer’s instruction. The luminescence signal was measured with a Tecan Spark® 10 M multiplate reader (Tecan) for 500 ms.

Data were normalized to the signal of DMSO treated cells. IC50 determination was performed using the Graph Pad Prims v. 7.03 ‘log (inhibitor) vs. response (three parameters)’ equation.

Synergism was analyzed by applying the Loewe [43] method implemented in Combenefit v. 2.02 [44].

### Extraction of total protein and Simple Western analysis

For total protein isolation, RIPA buffer (Cell Signaling Technology) containing protease- and phosphatase-inhibitor cocktails (Sigma-Aldrich) was used to lyse Dulbecco's phosphate-buffered saline (DPBS)-washed (Thermo Fisher Scientific) cell pellets on ice for 20 min. To remove debris, lysates were centrifuged at 4 °C and full-speed for 10 min. Afterward, the protein concentration was determined with the Pierce BCA Protein Assay Kit (Thermo Fisher Scientific).

If nothing else indicated, 0.2 mg/ml protein per 12–230 kDa capillary were used in a Simple Western analysis using the Wes instrument (ProteinSimple) as suggested by the manufacturer’s protocol. Antibodies against the following proteins were used in the indicated dilutions: CDH1 (#3195, Cell Signaling Technology, 1:1000 for 0.1 mg/ml protein), CDH2 (NBP1-48,309, Novus Biologicals, 1:100), ERK1/2 (#4695, Cell Signaling Technology, 1:50), HSP90 (sc-7947, Santa Cruz, 1:250 for 0.05 mg/ml and 0.2 mg/ml protein or 1:500 for 0.1 mg/ml protein), JUN (#9165, Cell Signaling Technology, 1:50), p-JUN (#9164, Cell Signaling Technology, 1:5), p-ERK1/2 (#4376, Cell Signaling Technology, 1:15), Vinculin (#13,901, Cell Signaling Technology, 1:30,000).

### Immunofluorescence

Murine cell lines grown to 80% confluency on chamber slides were fixed for 20 min in 4% paraformaldehyde (PFA) and permeabilized using 0.3% triton for 10 min at room temperature. After blocking in 5% BSA, the following primary antibodies were incubated for 1 h at room temperature: CDH1 (#3195, Cell Signaling Technology, 1:100), CDH2 (610,920, BD Bioscience, 1:200).

Fluorescent-labeled secondary antibodies (Thermo Fisher Scientific) were diluted 1:1000 and incubated together with 4′,6-diamidino-2-phenylindole (DAPI) for 1 h at room temperature protected from light.

Microscopy of the mounted slides was performed at an Axio Observer.Z1 (Carl Zeiss) using the indicated magnifications.

### Cytogenetic analysis

Parental cells of cell lines #3 and #9 were treated with colcemid for 4 h prior to harvest. Culture solution was centrifuged; the cell pellet was resuspended in a hypotonic 75 mM KCl-solution and incubated for 20 min at 37 °C. After centrifugation, cells were resuspended by dropwise adding 8 ml of an ice-cold fixative solution (3:1 mixture methanol and acetic acid). Cells were washed 3 times in 8 ml ice-cold fixative solution for 10 min each and dropped onto a fat-free and watered glass slide that was then air-dried overnight at 60 °C. Chromosomes were stained with Giemsa and examined under the microscope using 140 × magnification.

### Patient-derived xenografts

Already established patient-derived xenografts of pancreatic adenocarcinoma from three different male patients at passage number 2 were received from ARC-NET, University of Verona. At the time of surgery, patient 1 was 59 years old, patient 2 65 years and patient 3 53 years. Mice were maintained in the pathogen-free animal facility following institutional guidelines and with approval from the responsible authorities. The animals were housed under pathogen-free conditions in individually ventilated cages under standardized environmental conditions (22°C room temperature, 50 ± 10% relative humidity, 12 h light–dark rhythm). They received autoclaved food and bedding (Ssniff) and acidified (pH 4.0) drinking water ad libitum.

Tumor pieces of 3 mm^3^ were transplanted subcutaneously into NOD/SCID-mice with knocked IL2γ receptor (NSG mice) within 24 h after explant from donor mice. Remaining tumor tissue was preserved in DMSO or snap-frozen for later propagation or analyses. Engrafted tumors at a size of about 1 cm^3^ were surgically excised and fragments of 2–3 mm^3^ re-transplanted into immune deficient NMRI:nu/nu mice for further passage. Tumors were passaged not more than 10 times.

For drug screening studies, tumor material was implanted subcutaneously into appropriate cohorts of NMRI:nu/nu mice (n = 3 per treatment group). At advanced tumor size (200 mm^3^), mice were randomized and treated with 1 mg/kg trametinib (p.o., daily), 0.2 mg/kg decitabine (s.c., three times weekly). To further mouse cohorts, the combinations of trametinib and decitabine were applied. Tumor size was measured with a caliper instrument and monitored during the entire experiment with the measurements of two perpendicular tumor diameters using the spheroid equation: tumor volume = [(tumor width)^2^ × tumor length] × 0.5. Treatment was continued over a period of two weeks unless tumor size exceeded 10% of animal body weight or animals showed loss of more than 15% body weight. Six hours after last treatment animals were sacrificed and tumor samples preserved for further analyses.

### Mass spectrometry

#### Sample preparation

Cell pellets of parental, resistant and P12 cells of six different lines were resuspended in 100 μl 50 mM ammonium bicarbonate and 0.1% sodium deoxycholate (NaDOC) for cell lysis. Samples were sonicated on ice for 10 min and centrifuged (16,000 g, 15 min, 4°C). Protein concentration was determined via Bradford assay. Due to a very low concentration, technical replicates were pooled. The samples were ridded of remaining viscosity with 10 impulses at 5% power by ultrasonic homogenization via Sonopuls HD 200 MS 72 (Badelin) and centrifuged (16,000 g, 15 min, 4°C). Protein amount was determined via amino acid analysis. DTT (5 mM) was added to the sample for reduction (30 min, 60°C), followed by iodoacetamide (15 mM) for alkylation (30 min, room temperature, in the dark). Lysed proteins were tryptically digested over night at 37°C (trypsin/protein ratio 1/24). For acidification, trifluoroacetic acid (TFA) (0.5%) was added (30 min, 37°C), samples were centrifuged (10 min, 16,000 g) for removal of NaDOC and supernatant transferred to glass vials, dried in a vacuum centrifuge, and dissolved in 0.1% TFA. A sample amount corresponding to 275 ng was used for one liquid chromatography tandem mass spectrometry (LC–MS/MS) measurement.

#### LC–MS/MS parameters

LC–MS/MS analysis was performed on a LTQ Orbitrap Elite instrument (Thermo Fisher Scientific) coupled online to an upstream-connected Ultimate 3000 RSLCnano high-performance liquid chromatography system (Dionex). Samples were measured in a shuffled manner. Peptides dissolved in 0.1% TFA were pre-concentrated on a C18 trap column (Acclaim PepMap 100; 100 μm × 2 cm, 5 μm, 100 Å; Thermo Fisher Scientific) within 7 min at a flow rate of 30 μl/min with 0.1% TFA. Peptides were then transferred to an in-house packed C18 analytical column (ReproSil®-Pur from Dr. Maisch HPLC GmbH, Ammerbuch, Germany, 75 μm × 40 cm, 1.9 μm, 120 Å). Peptides were separated with a gradient from 5 to 40% solvent B over 98 min at 300 nl/min and 65°C (solvent A: 0.1% formic acid; solvent B: 0.1% formic acid, 84% acetonitrile). Full-scan mass spectra in the Orbitrap analyzer were acquired in profile mode at a resolution of 60,000 at 400 m/z and within a mass range of 350–2000 m/z. MS/MS spectra were acquired in data-dependent mode at a resolution of 5,400. For MS/MS measurements, the 20 most abundant peptide ions were fragmented by collision-induced dissociation (normalized collision energy (NCE) of 35) and measured for tandem mass spectra in the linear ion trap.

#### Protein identification and quantification

Proteins were identified with Proteome Discoverer v. 1.4 (Thermo Fisher Scientific). Spectra were searched against the UniProtKB/Swiss-Prot database (Release 2018_11; 53,780 entries) using Mascot v. 2.5 (Matrix Science, London, UK). Taxonomy setting was *Mus musculus*, and mass tolerance was 5 ppm and 0.4 Da for precursor and fragment ions, respectively. Dynamic and static modifications were considered for methionine (oxidation) and cysteine (carbamidomethyl), respectively. The FDR was calculated with the Proteome Discoverer Target Decoy PSM Validator function, and identifications with a FDR > 1% were rejected. The software Progenesis QI v. 2.0.5387.52102 (Nonlinear Dynamics) was used for label-free quantification. The obtained raw files were aligned to a reference run and a master map of common features was applied to all experimental runs to adjust for differences in retention time. Ion charge states of 2 + , 3 + , and 4 + with a minimum of three isotope peaks were considered, and raw ion abundances were normalized for automatic correction of technical or experimental variations between runs. Quantified features were identified using the obtained Proteome Discoverer identifications. All non-conflicting peptides were considered for protein quantification.

#### Statistics

Normalized protein abundances were obtained from Progenesis and analyzed by applying ANOVA followed by Tukey’s honest significant difference (HSD) method. Fold changes between groups were determined based on normalized abundances, while ANOVA was calculated using arcsinh-transformed data for consistency with the Progenesis QI software. The FDR was controlled by adjusting ANOVA *p* values using the method of Benjamini and Hochberg [45]. For proteins with adjusted ANOVA *p* values below the significance level of α = 0.05, the TukeyHSD method was applied to further characterize the identified differences in abundance levels between groups. Proteins were considered differentially abundant between groups with a log_2_ fold change ≥ 1 or ≤ − 1 and an adjusted p value < 0.05.

### Isolation of nucleic acids

DNA and RNA were isolated using the Maxwell® RSC Cultured Cells DNA and the Maxwell® RSC simplyRNA Cells Kit (Promega) according to manufacturer’s instruction. Nuclease-free water was used for DNA elution.

### RNA-sequencing

#### Sequencing

RNA-sequencing of parental, resistant and P12 cells of six different cell lines was performed by CeGaT (Tübingen). In addition, P5 cells of cell lines #3, #7 and #9 were sequenced. For library preparation, the TruSeq Stranded mRNA Kit (Illumina) was used with 100 ng input RNA and 2 × 100 bp were sequenced on a HiSeq 4000 (Illumina) or a NovaSeq 6000 system (Illumina).

#### Read processing and quantification

Demultiplexing of the sequencing reads was performed with Illumina CASAVA v. 2.17 or bcl2fastq v. 2.19. Adapters were trimmed with Skewer v. 0.1.116 or 0.2.2 [46].

Transcripts were quantified using the quasi-mapping approach of salmon v. 0.12 [47]. TXImport v. 1.6 [48] and DESeq2 v. 1.18 [49] were used to import transcript-level counts, convert them to gene-level counts and perform differential expression analysis between all four cell states (parental, resistant, P5, P12). Results were multiple test-corrected by the Benjamini–Hochberg method.

#### Principal component analysis

Principal component analysis (PCA) was performed on the normalized gene-level counts of all expressed genes.

#### Hierarchical clustering

Hierarchical clustering of significantly differentially expressed genes (Benjamini–Hochberg adjusted *p* value < 0.01; log_2_ fold change > 1 or log_2_ fold change < − 1) between parental versus the union of resistant, P5, P12 and resistant versus the union of parental, P5, P12 was computed by the ward.D2 method. Additionally, the same method was used to cluster samples based on PDAssigner genes [28] or PDAC subtype associated genes defined by Bailey et. al. [27] and Moffitt et al. [29].

#### Gene set enrichment analysis

GSEA [50] was performed using default settings and gene set permutation.

#### Score to define reverting Transcripts

In order to identify differentially expressed genes that show a similar expression pattern in parental and P12 samples, a score was defined based on the log_2_ fold change between parental/P12 and resistant samples.

The score was defined as follows:

$$s = \left\{ {\begin{array}{*{20}c} 0 & {{\text{if}} - 1 < \log_{2} FC(P,R) < 1} \\ 0 & {{\text{if}}\;\log_{2} FC(P,R) \cdot \log_{2} FC(P12,R) < 0} \\ {\frac{{\max (|\log_{2} FC(P,R)|,|\log_{2} FC(P12,R)|)}}{{\min (|\log_{2} FC(P,R)|,|\log_{2} FC(P12,R)|)}}} & {{\text{otherwise}}} \\ \end{array} } \right.$$

where $log_{2} FC(A,B)$ describes the log_2_ fold change between A and B. Genes were only considered if the log_2_ fold change between parental and resistant cell state was reasonably large.

### Whole genome bisulfite sequencing

#### Sequencing and alignment

Whole genome bisulfite sequencing (WGBS) of parental, resistant and P12 cells of cell lines #3 and #9 was performed at the Genomics and Proteomics Core Facility of the German Cancer Research Center (GPCF DKFZ, Heidelberg) using the TruSeq DNA PCR-free Methyl protocol (Illumina) for library preparation. A HiSeq X machine (Illumina) was used for 150 bp paired-end sequencing. Reads were mapped using bwa-meth v. 0.2.0 [51] on the GRCm38 assembly with added PhiX genome as a sequencing control.

#### Calculation of methylation levels

CpG methylation levels were computed using an in-house script filtering reads with a mapping quality < 30 and bases with base quality < 17.

#### Differentially methylated region detection

The BSmooth algorithm of bsseq v. 1.10 [52] was used to detect DMRs between parental and resistant samples common both for cell line #3 and #9, with every DMR containing a minimum of four CpGs and a minimum difference in methylation level of 0.4.

#### Nearest genes

For each DMR, the nearest flanking genes were determined by finding the nearest TSSs to each region using BEDTools closest v. 2.27 [53].

#### Integration with RNA-seq data

Expression changes of the two nearest genes of every DMR were assessed from RNA-sequencing data as described above. Genes with a log_2_ fold change > 1 were defined as upregulated in resistant cells, those with a log_2_ fold change < − 1 as downregulated in resistant cells.

#### Genomic regions

The localization of DMRs relative to genes and CpG islands was performed using BEDTools intersect v. 2.27 (Quinlan and Hall, 2010). Reference data were taken from Ensembl build 93 [54] (genes) and the UCSC database (CpG islands).

Reference data for shore and shelf regions were created using BEDTools flank v. 2.27 [53]; shores were defined as regions up to 2000 bp away from CpG islands and shelfs as regions up to 2000 bp away from shores.

To test for significance of the association between the genomic regions and the DMRs compared to the mm10 reference genome (GCF_000001635.20), the chi-square test was applied.

#### Methylation score to define reverting DMRs

A score was used to model the methylation changes between parental, resistant and P12 cells, where scores > 0.5 indicate that the P12 methylation is closer to the parental level, scores < 0.5 indicate the P12 methylation is closer to the resistant level.

$$s = \left\{ \begin{gathered} 0,\quad \quad \quad \quad \quad {\text{if }}\Delta (P,R) \cdot \Delta (P,P12) < 0 \hfill \\ 1,\quad \quad \quad \quad \quad {\text{if }}\Delta (P,R) \cdot \Delta (R,P12) < 0 \hfill \\ \frac{|\Delta (P,P12)|}{{|\Delta (P,R)|}},\;{\text{otherwise}} \hfill \\ \end{gathered} \right.$$

where $\Delta (A,B)$ describes the difference in methylation between A and B. A cut-off of 0.44 (90% quantile) was used to define DMRs as reverting.

#### Conservation

The UCSC liftOver tool was used together with the mm10ToHg38 liftOver chain in order to identify regions in the human genome that are associated with the murine DMRs. The minimum ratio of bases that need to remap to define a region as valid liftOver was set to 0.5.

#### Regulatory regions

Overlaps between DMRs and transcription factor binding sites, miRNA target regions and VISTA enhancers were computed using BEDTools intersect v. 2.27 [53]. Reference data were taken from the Ensembl regulation build 93 [54].

The findMotifsGenome script from homer v. 4.9 was used in order to find enrichment of known binding motifs from the homer library [55]. The script was used with standard parameters (-size 200 -cpg) on the GRCm38 assembly, comparing reverting DMRs to random background sequences.

#### Overlap with chromatin marks from PDAC organoids

Organoid data available from Roe et al. [34] were used to check whether DMRs overlap with open chromatin or enhancer regions.

Analysis was performed on the following organoids.

H3K27ac-ChIP-seq:

- N5, N6—normal pancreatic organoids [56]
- T3, T6, T19, T23, T33, T34—tumor organoids

ATAC-seq:

- N5, N6—normal pancreatic organoids [56]
- T3, T6, T23—tumor organoids

Reads were aligned with BWA-MEM v. 0.7.17 [57] against the GRCm38 genome. Duplicate reads were further removed using Samtools v. 1.9.

Identification of ChIP-seq and ATAC-seq peaks was performed using MACS2 v. 2.1.2 [58, 59] callpeak function with default settings. Resulting narrowPeak files were further merged with BEDtools merge v. 2.27 [53] and overlapped with the DMRs using BEDtools intersect to analyze how many peaks from tumor and normal organoids are located within the DMRs separately for H3K27ac ChIP-seq and ATAC-seq peaks.

#### Enrichment analysis

In order to detect possible enrichment of TFBS as well as ATAC-seq and ChIP-seq peaks in DMRs compared to the remaining genome, every DMR was matched with 1 million randomly picked genomic regions with similar length and CpG count. The occurrence of the features of interest was then compared between DMRs and the average of the randomly chosen regions. The occurrence of the features of interest was then compared between DMRs and the average of the randomly chosen regions. A feature was defined as significantly enriched if its occurrence in the DMRs was larger than in the average of random regions in at least 95%, 99% or 99.9% of all comparisons (significance level < 0.05, < 0.01 or < 0.001).

### Targeted deep bisulfite sequencing

Targeted deep bisulfite sequencing was performed as described elsewhere [33]. DMRs for validation were selected based on the following criteria: Proportion of hypermethylated DMRs reflects that of identified DMRs (> 90%), reversion in P12, conserved in humans, preferentially DMRs with at least one flanking transcript showing reverted expression in P12. Primer sequences are listed in Additional file 3: Table S1. The MiSeq (Illumina) run was conducted by the BioChip-Laboratory of the Essen University Hospital. Amplikyzer2 v. 1.2.0 was used for analysis. Due to the much higher coverage compared to WGBS, DMRs were classified as positively validated at a minimum methylation difference of 0.2.

### Whole genome sequencing

#### Sequencing and genome mapping

The Genomics and Proteomics Core Facility of the German Cancer Research Center (GPCF DKFZ, Heidelberg) performed the library preparation for WGS of parental and resistant cells of cell lines #3 and #9 as well as a control tail samples corresponding to line #3 with the TruSeq DNA PCR-free Kit (Illumina) and the 150 bp paired-end sequencing on a HiSeq X (Illumina).

Reads were aligned to the mouse reference genome GRCm38 using BWA-MEM v. 0.7.15 [57] with default settings. Duplicate reads were marked with sambamba v. 0.6.5 [60].

#### Variant calling

Variants and small insertions and deletions (InDels) were called in a two-step process. First, candidate variants were called using freebayes v. 1.1.0 [61] with parental and resistant tumor samples as well as a normal tail tissue.

In a second step, variants were validated and readjusted using Varlociraptor v. 1.1.1 [62]. The validated variants were separated into different groups according to their change in VAF between parental and resistant samples. Variants were defined as present in parental (VpPs) if they drop to a VAF of 0 from parental to resistant. Variants with a VAF > 0.1 in resistant and a VAF of 0 in parental were defined as present in resistant (VpRs). Variants with a VAF > 0 in both parental and resistant tumor samples were defined as present in parental and resistant (VpPRs).

VpPs and VpRs were validated in P5 and P12 tumors using the available WGBS data. Since the technical differences between WGBS and WGS affect the comparability of results from both methods, validation was performed solely on WGBS samples. VAFs in WGBS samples were called using Varlociraptor v. 1.1.1 [62], with VpPs and VpRs from WGS as candidate variants. To adjust for bisulfite conversion, only A > T and T > A variants covered > 15 × were kept for WGBS validation.

#### Data management and annotation

Snakemake v. 5.10 [63] was used as workflow management system for the complete computational analysis.

Data management and visualization was performed using bcftools v. 1.9 [64] and python v. 3.7 libraries seaborn v. 0.9 and pandas v. 0.24.

Variants were annotated using Jannovar v. 0.25 [31] with the GRCm38 annotation database as well as SIFT scores (Ng and Henikoff, 2003) (Download source: http://sift.bii.a-star.edu.sg/sift4g/public//Mus_musculus/GRCm38.83.zip).

To compare SNV positions between both mouse lines, the closest variant positions between both variant calls were identified using BEDtools closest v. 2.27 [53].

## Statistics

Replicates were performed as indicated in the figure legends. For statistical analyses R v. 3.6.0 [65] and GraphPad Prism v. 7.03 were used. The applied test is described in the figure legends, respectively.

### Supplementary Information

**Additional file 1**. Supplementary Tables 3–5, 7

**Additional file 2**. Supplementary Figures.

**Additional file 3**. Supplementary Tables 1, 2, 6.

## Data Availability

Proteomics data have been deposited as a complete submission in the ProteomeXchange Consortium via the PRIDE partner repository (http://www.proteomexchange.org; data set identifier: PXD018093 and 10.6019/PXD018093). The.msf files obtained in Proteome Discoverer were converted into the mzIdentML standard format using ProCon PROteomics Conversion tool version 0.9.718 (PubMed ID 26182917). The RNA-seq generated during this study is available at GEO: GSE146348. The accession number for WGBS and WGS data deposited on ENA is: PRJEB37018. The custom code generated and used during the current study is available from the corresponding author on request.

## Abbreviations

ATAC-seq: Assay for transposase-accessible chromatin and sequencing
BET: Bromodomain and extra-terminal motif
ChIP-seq: Chromatin immunoprecipitation and sequencing
CpG: Cytosine-guanine dinucleotide
DMEM: Dulbecco's Modified Eagle medium
DMR: Differentially methylated region
DMSO: Dimethyl sulfoxide
DNMT_i_: DNA methyltransferase inhibitor
DPBS: Dulbecco’s phosphate-buffered saline
EDTA: Ethylenediaminetetraacetic acid
FDR: False discovery rate
GEMM: Genetically engineered mouse model
GSEA: Gene Set Enrichment Analysis
HDAC_i_: Histone deacetylase inhibitor
HSD: Honest significant difference
kde: Kernel density estimation
LC: Liquid chromatography
MEK: Mitogen-activated protein kinase
MEK_i_: Mitogen-activated protein kinase inhibitor
miRNA: Micro RNA
MS: Mass spectrometry
NaDOC: Sodium deoxycholate
PDAC: Pancreatic ductal adenocarcinoma
PDX: Patient-derived xenografts
PI: Propidium iodide
PI3K: Phosphatidylinositol 3-kinase
P5: Passage 5 after drug withdrawal
P12: Passage 12 after drug withdrawal
P40: Passage 40 after drug withdrawal
RNA-seq: RNA-sequencing
SAHA: Suberoylanilide hydroxamic acid
SD: Standard deviation
SEM: Standard error of the mean
SNV: Single nucleotide variant
TES: Transcription end site
TFA: Trifluoroacetic acid
TFBS: Transcription factor binding sites
TRE: 12-O-tetradecanoylphorbol-12-acetate response elements
Trp53: Transformation related protein 53
TSS: Transcription start site
VAF: Variant allelic fraction
VpP: Variant present in parental
VpPR: Variant present in parental and resistant
VpR: Variant present in resistant
WGBS: Whole genome bisulfite sequencing
WGS: Whole genome sequencing
