# Supplementary material for: Pancreatic cancer acquires resistance to MAPK pathway inhibition by clonal expansion and adaptive DNA hypermethylation
Source: Clin Epigenetics. 2024 Jan 16;16:13. doi: 10.1186/s13148-024-01623-z (PMC10792938; doi:10.1186/s13148-024-01623-z)
Supplement: Supplementary file 2 — Additional file 2. Supplementary Figures. [file 13148_2024_1623_MOESM2_ESM.pdf]

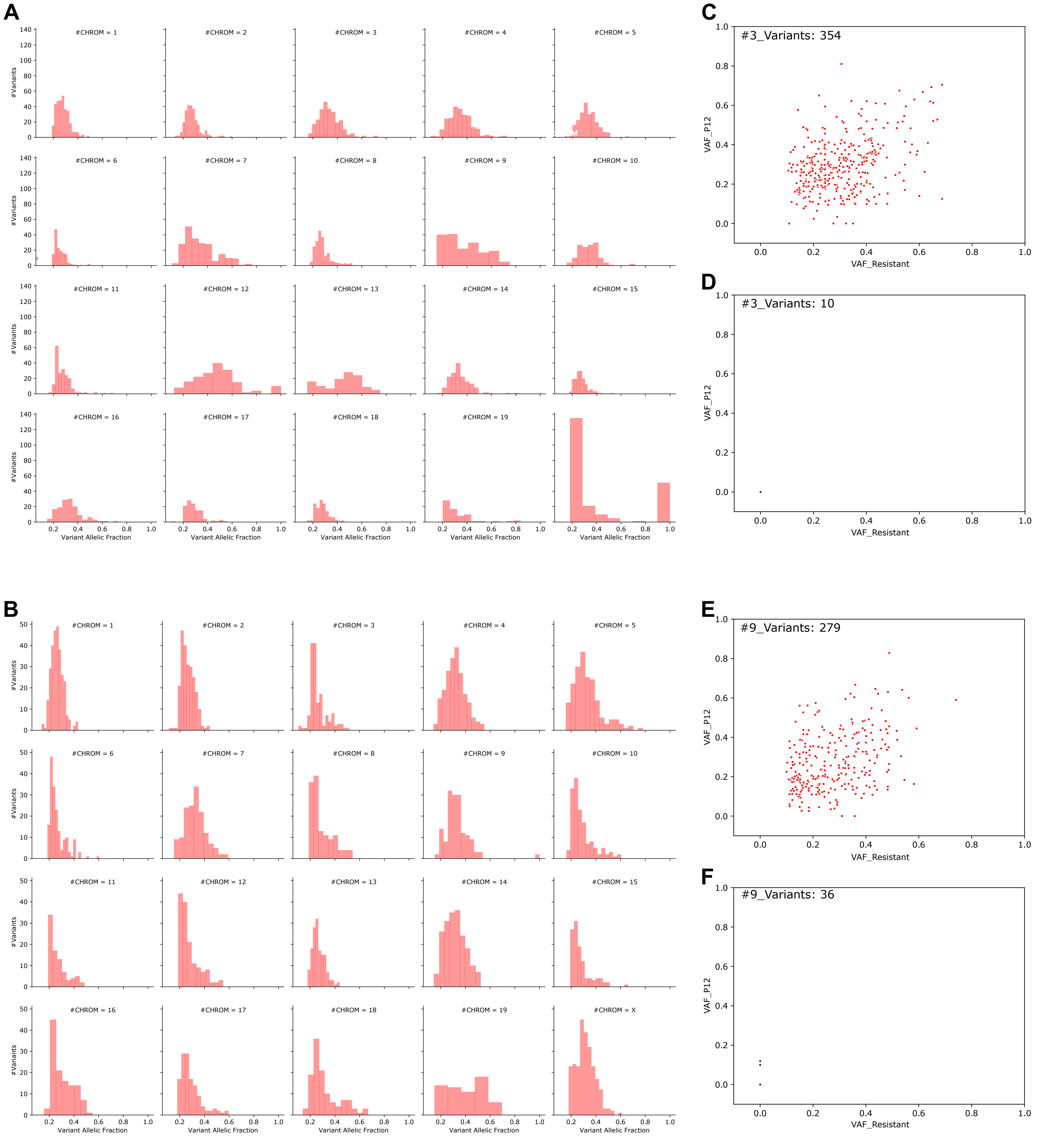

Supplementary Fig. 1

VAF of VpRs called by WGS separated by chromosomes for cell lines #3 **(A)** and #9 **(B)**. **(C,E)** VAF of VpRs in resistant compared to P12 cells displayed for cell lines #3 **(C)** or #9 **(E)**. **(D,F)** Scatter plot for the VAF of VpPs in resistant compared to P12 in cell lines #3 **(D)** or #9 **(F)**. Only A > T and T > A variants called by WGS and validated by WBGS are shown.

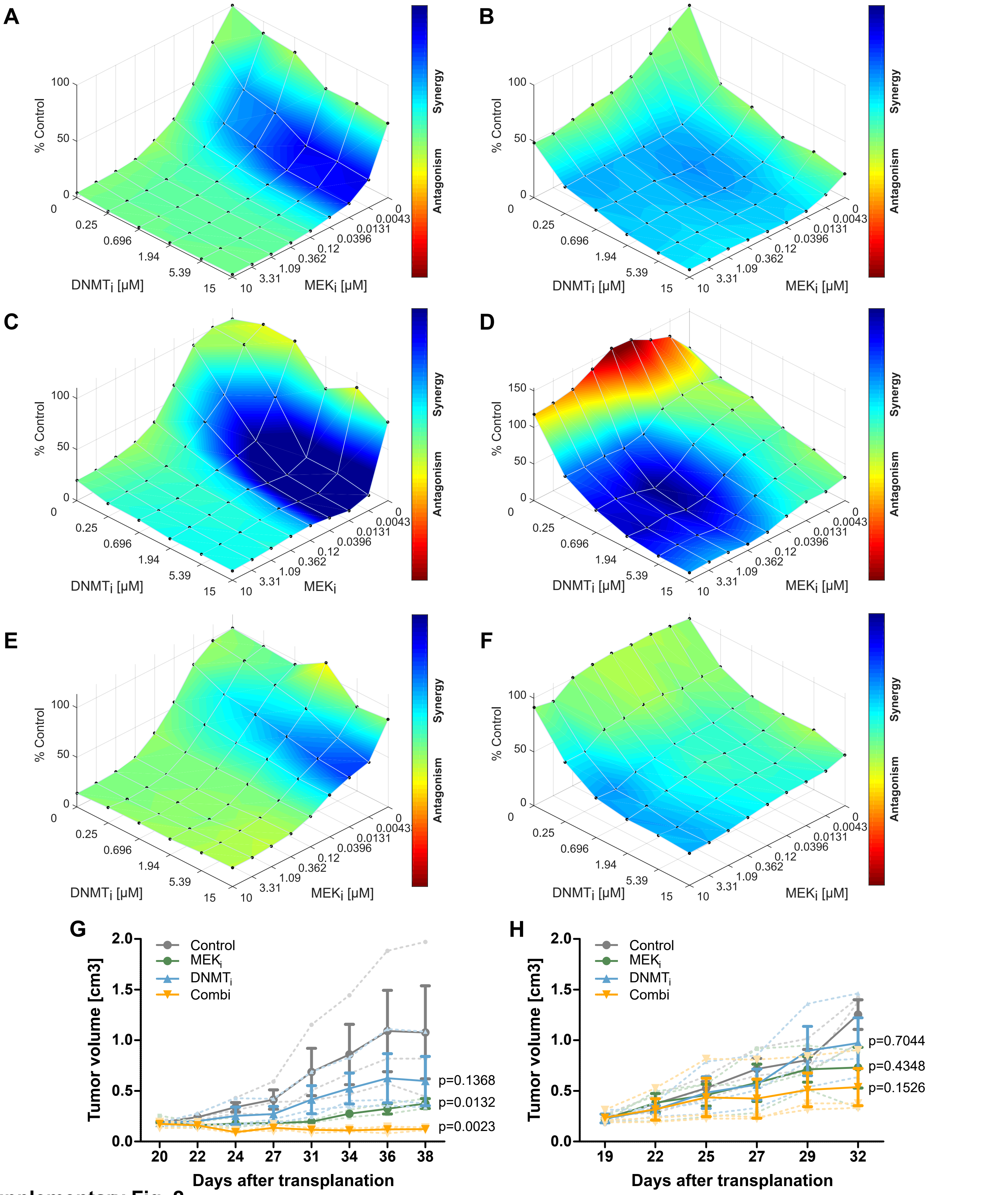

Supplementary Fig. 2

(A-F) Synergy analysis of MEK_i plus $DNMT_i$ using the Loewe method of the Combenefit software shown for cell lines #4 **(A,B)**, #9 **(C,D)** and #10 **(E,F)**. Results for parental cells are shown left, while resistant cells are displayed in the right panels, respectively. **(G,H)** Two different PDX of PDAC treated either with MEK_i, $DNMT_i$ or the combination. Solid lines represent the mean tumor volume of the three mice per treatment group ± SEM.

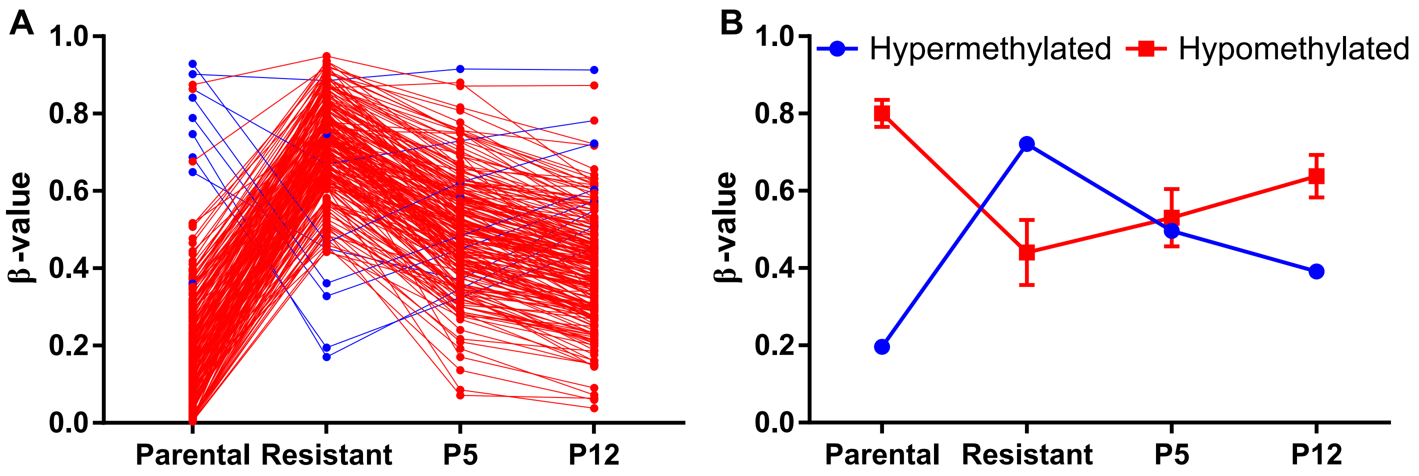

Supplementary Fig. 3

(A) Methylation pattern of 217 reverting DMRs divided into hypo- (blue) and hypermethylated (red) regions. **(B)** Mean DNA methylation of 217 reverting DMRs ± SEM separated into hypo- and hypermethylated regions.

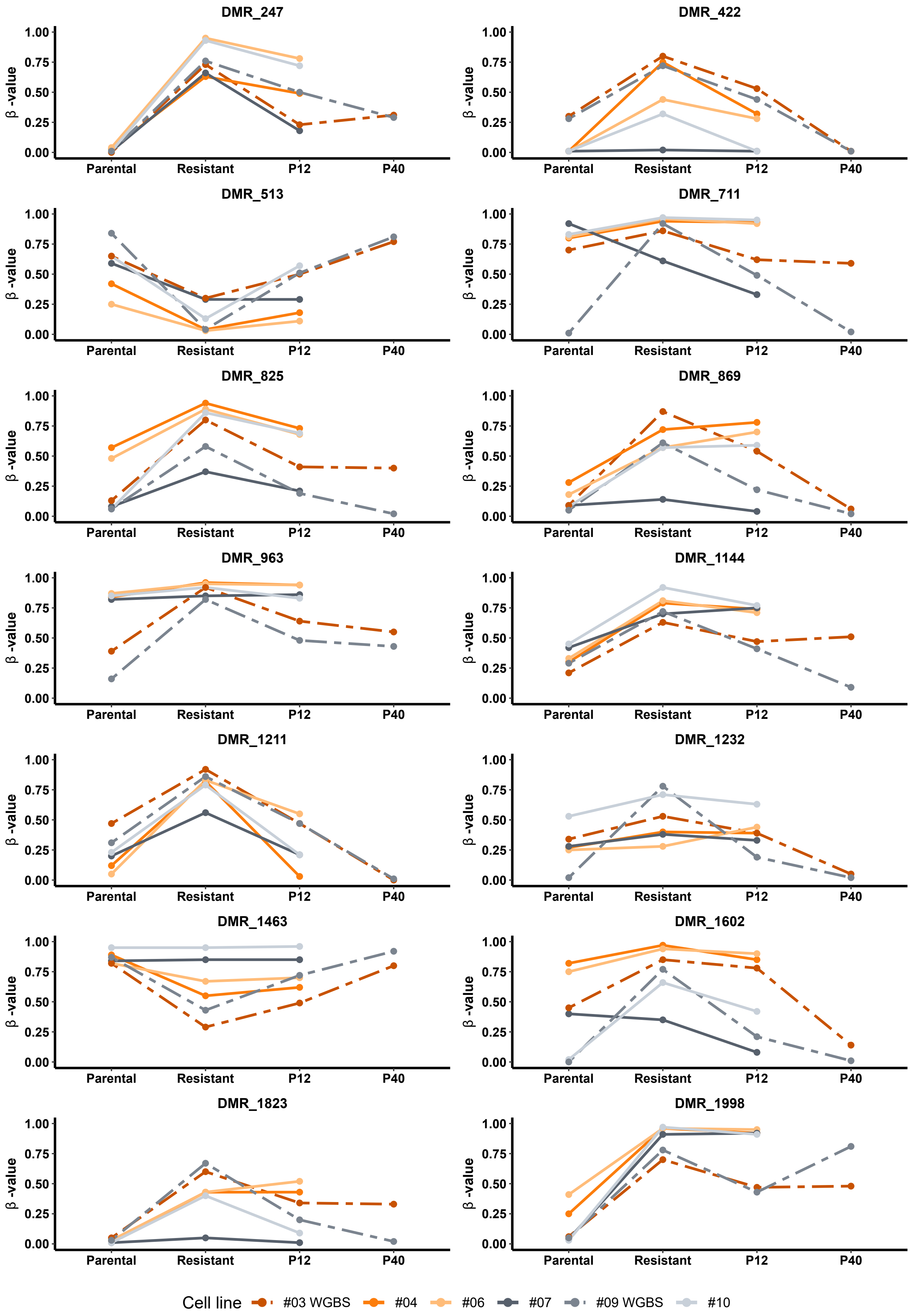

Supplementary Fig. 4

Individual methylation patterns of 14 DMRs validated by targeted deep bisulfite sequencing in four independent cell lines compared to cell lines #3 and #9 analyzed by WGBS. In addition, P40 of #3 and #9 was measured by targeted deep bisulfite sequencing. DMR_929 is displayed in Fig. 4D,E.

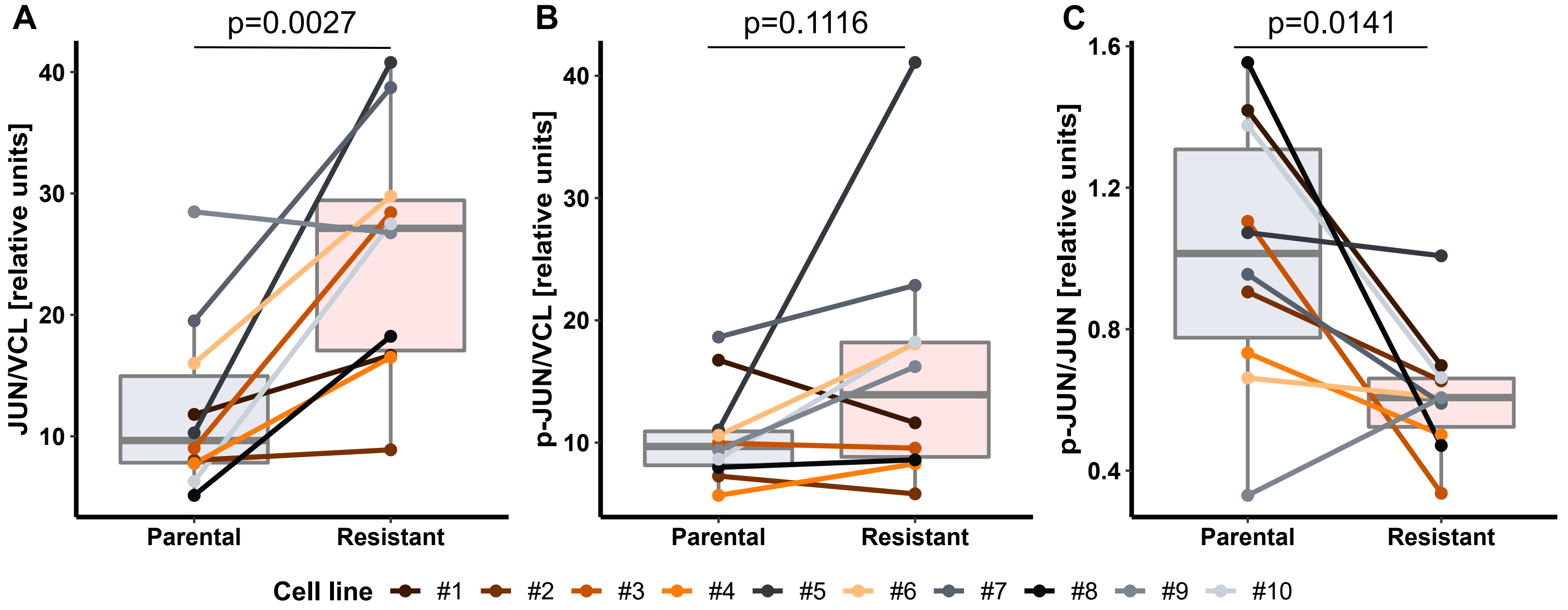

Supplementary Fig. 5

Relative protein expression of total JUN **(A)** or p-JUN **(B)** compared to VCL in parental and resistant cells. **(C)** Proportion of p-JUN to JUN. Statistics were calculated using the two-tailed paired Student's t-test.
