# Supplementary material for: Pancreatic cancer acquires resistance to MAPK pathway inhibition by clonal expansion and adaptive DNA hypermethylation
Source: Clin Epigenetics. 2024 Jan 16;16:13. doi: 10.1186/s13148-024-01623-z (PMC10792938; doi:10.1186/s13148-024-01623-z)
Supplement: Supplementary file 3 — Additional file 3. Supplementary Tables 1, 2, 6. [file 13148_2024_1623_MOESM3_ESM.pdf]

Supplementary Table 1

Primer sequences used for targeted deep bisulfite sequencing of selected differentially methylated regions (DMRs). During oligonucleotide synthesis the following tag sequences were added to the 5´end of each forward and reverse primer: CTTGCTTCCTGGCACGAG (forward) and CAGGAAACAGCTATGAC (reverse)

DMR	Primer	Sequence	PCR product location in mm10
247	forward	ATTTTATGGGTTAGTTGTTTGATTT	chr10:99266158-99266419
	reverse	CAACTAATAACACTTTTAACACCC	
422	forward	GTTTGTTTGTAATTGGGGATTAGG	chr11:113060998-113061290
	reverse	TCTAAAATACAACCCTACATATAAATC	
513	forward	TTGTTAAATGGAATTGTTGTTGAGA	chr12:85859330-85859634
	reverse	AAAACTCTTACATAAACTACCAAAC	
711	forward	TAAAGTAGTGGGATAAATTTTTTTT	chr15:27467984-27468252
	reverse	CTAATCTTCTTATTCTTAACAAATCC	
825	forward	AGATTAGTGTTTTTAATTATTTTGA	chr16:23272009-23272213
	reverse	CCAAACAAAAAACATCATACTTTC	
869	forward	TTTTTTGTTTAAGGAAAGGATA	chr16:92620511-92620772
	reverse	CCATTCTCAAAACAAATTTTAC	
929	forward	TTGTAAGGTTGTATTTTTTTTGATGTTT	chr17:44718034-44718321
	reverse	CAACCCTTAAATACTAAAACTCAACTC	
963	forward	TGGGAGGTAGTGTGGGATATAGTAG	chr17:84141583-84141880
	reverse	CTTCCCAAAAACAAAAACACTCTAA	
1144	forward	AGGAGTGTGTTAATTTTTAGGGGTTA	chr2:26501667-26501948
	reverse	CCCAACCAAATAAAACCTACCTAA	
1211	forward	ATTTGTTAGTATAGAAGAAGGTTGGTAGT	chr2:102873250-102873543
	reverse	AACAAATTCTAAAAAAAATTCCCTC	
1232	forward	TTTGTTTAGGTTTTGTTTTTTGTTG	chr2:127336828-127337044
	reverse	ATTCACACCATTTACAAATCACAC	
1463	forward	GTTGATTATTAGTTTTTTTTGTAGTT	chr4:119244934-119245233
	reverse	CACATTTACACTAATATCCCAACC	
1602	forward	TAGTAATATAAGATGTTTAAGTATTGAA	chr5:98943301-98943589
	reverse	AAAACCCCACAAAACTCTCTCTAA	
1823	forward	AAAAGTTTTAGAGTGAGTAGAATAGGT	chr7:24370317-24370596
	reverse	CTTACAACCCAAAAACCACAAC	
1998	forward	TAATTTTAGTATATGGGGAGTGGGA	chr8:34813921-34814236
	reverse	TAACCATTCCCTTAAAAACACTACC	

Supplementary Table 2

IC50 of trametinib in the parental cells based on averaged dose response curves.

Cell line	IC50 [nM]	Replicates
#1	25.2	2
#2	9.4	2
#3	10.9	3
#4	11.2	3
#5	12.9	2
#6	16.3	3
#7	14.3	3
#8	5.3	2
#9	55.2	3
#10	10.9	3

Supplementary Table 6

Numbers (No.) of evaluated mitosis are displayed (NA, not available).

No._mitosis	Karyotype_cell line #3	Karyotype_cell line #9
1	76	70
2	79	70
3	78	70
4	74	67
5	NA	59
6	NA	70
7	NA	70
8	NA	60
9	NA	73
10	NA	70
11	NA	63
12	NA	70
